# Analysis of SAW Temperature Properties in KTiOPO_4_ Single Crystal

**DOI:** 10.3390/ma16010069

**Published:** 2022-12-21

**Authors:** Rinat Taziev, Victor Atuchin

**Affiliations:** 1 Laboratory of Optical Materials and Structures, Institute of Semiconductor Physics, SB RAS, 630090 Novosibirsk, Russia; 2 Research and Development Department, Kemerovo State University, 650000 Kemerovo, Russia; 3 Department of Industrial Machinery Design, Novosibirsk State Technical University, 630073 Novosibirsk, Russia; 4 R&D Center “Advanced Electronic Technologies”, Tomsk State University, 634034 Tomsk, Russia

**Keywords:** KTiOPO_4_, piezoelectric, surface acoustic wave, temperature coefficient of delay

## Abstract

The surface acoustic wave (SAW) properties of potassium titanyl phosphate (KTiOPO_4_, KTP) single crystal were evaluated by numerical methods. The phase velocity, electromechanical coupling coefficient, power flow deflection angle, and temperature coefficient of delay (TCD) were determined for different crystal cuts of KTP. It was shown that SAW has the electromechanical coupling coefficient of 0.59% and the TCD of 62 ppm/°C on the Z-cut and wave propagation direction along the crystal X + 70°-axis. For the Z-cut and wave propagation direction along the X-axis, the pseudo-surface wave (PSAW) is characterized by the coupling coefficient of 0.46% and the TCD value of 57 ppm/°C. The Bleustein–Gulyaev (BG) wave has the TCD value of 35 ppm/°C and 41 ppm/°C on the Y- and X-cuts of KTP, respectively.

## 1. Introduction

Potassium titanyl phosphate KTiOPO_4_ (KTP) is a ferroelectric crystal commonly used for the frequency doubling of Nd^3+^-doped laser sources. KTP is of great significance in the field of nonlinear optics due to its large nonlinear coefficients and a wide transparency range [1,2,3,4,5]. KTP crystals are used in the devices for the conversion of IR radiation to the THz wave and THz radiation to the THz wave [5]. The generation of THz wave is a parametric process in KTP, and this material has great advantages in comparison with LiNbO_3_:MgO crystals. In the KTP structure, wide-range substitutions are possible in cation and anion sublattices, and a great diversity of crystals is presently known in the KTP family [6,7,8,9,10,11,12]. Accordingly, a significant tuning of physical properties can be reached via a solid solution formation. It is known that KTP-type crystals are characterized by high thermal and chemical stabilities [6,9,13,14]. Presently, large-size monodomain KTP crystals of high optical quality and high electrical resistivity are available for industrial applications [15,16,17].

As a multifunctional material, KTP possesses piezoelectric properties, which can be useful for SAW device applications. The surface acoustic wave excitation by an interdigital transducer (IDT) in KTP was first experimentally investigated in [18]. A full set of elastic, piezoelectric, and dielectric constants of KTP was established in [19,20]. It was shown that the pseudo-surface wave and the Bleustein−Gulyaev (BG) wave are excited on Z- and Y-cuts, respectively, for the wave propagation direction along the X-axis in KTP [21]. The pseudo-surface acoustic wave is similar to the Rayleigh wave because it has no transverse displacement component. BG waves, due to their high sensitivity to fluid viscosity, are of considerable interest for the use in biomedical applications [22,23,24,25,26]. For the BG wave propagation, the displacement of material particles occurs only parallel to the substrate surface (there is no normal component), and it prevents its penetration into a liquid on the surface. Therefore, the BG wave cannot be used for microfluidic excitation, and it makes them suitable for measuring a liquid viscosity. The surface acoustic wave is widely used in the temperature, pressure sensors, and microfluidic applications [27,28,29,30,31,32,33]. Generally, the SAW has three components of mechanical displacement on the crystal surface: the first one is parallel to the direction of wave propagation, the second one is perpendicular to the direction of propagation, and the third one is normal to the substrate surface. Typically, the magnitude of the SAW amplitude reaches from a fraction to several nanometers, and it decreases exponentially with the depth into the crystal. In the layer with a thickness of about one wavelength, almost all the wave energy is concentrated, and in such a way that the SAW is strongly localized near the substrate surface. In addition, since the wave has both longitudinal and transverse displacement components, it can effectively “sense” the properties of an adjacent medium (gas, liquid, solid films, small particles, etc.). For microfluidic applications, the normal displacement component is important, since the Rayleigh wave penetrates a liquid drop on the substrate surface, thus, transforming into a leaky wave. The leaky wave creates the acoustic pressure in a liquid medium, which can cause the liquid drop movement called acoustic streaming, that forms the basis for many microfluidic devices [34,35,36,37,38,39,40,41,42,43]. To study the effects of the temperature change on the propagation of SAW, pseudo-SAW and BG waves, the dependence of material constants on temperature should be determined. This allows the temperature sensor creation on the base of SAW in many microfluidic devices.

Recently, the temperature dependence of the piezoelectric constants of KTP was determined with the use of single sample by the method of resonant ultrasound spectroscopy (RUS) [44]. It utilizes a wide resonance spectrum of bulk acoustic eigen modes excited in one cubic sample, containing all the necessary information on KTP material constants. However, at present, the characteristics of the surface, pseudo-surface and Bleustein–Gulyaev waves and their temperature properties are not studied in detail numerically or experimentally on KTP.

This work is aimed at the numerical study of the temperature properties of surface acoustic waves in KTP. For generality, it is also necessary to investigate such important SAW parameters as the phase velocity, electromechanical coupling coefficient, and power flow deflection angle on X-, Z-, and Z-rotated cuts of KTP. The paper is organized as follows: (1) The contour plots of the main SAW characteristics (phase velocity, electromechanical coupling coefficient, power flow deflection angle, and temperature coefficient of delay) are simulated by changing Euler angles. (2) For selected Z- and X-cuts of the KTP crystal, the SAW parameters are investigated in detail to find the SAW propagation directions which can be of potential interest for SAW device applications. (3) FEM simulations are performed in the time domain to investigate the selected SAW, pseudo-SAW, and Bleustein–Gulyaev wave propagation orientations in KTP.

## 2. Materials and Methods

KTP belongs to the orthorhombic crystal system (mm^2^ point group), and it has 17 independent material constants necessary to simulate its acoustic properties: nine elastic constants, five piezoelectric, and three dielectric constants. In our numerical simulations, all necessary material constants of the KTP crystal were taken from [19,20]. All these constants (elastic stiffness tensor C^E^_ij_, piezoelectric stress tensor e_ij_, and dielectric constants ε^S^_ij_) can be defined as the matrix elements [19,20]:

$$C^{E}=\left(\begin{array}{cccccc} 1.70 & 0.333 & 0.40 & 0 & 0 & 0\\ 0.333 & 1.74 & 0.35 & 0 & 0 & 0\\ 0.40 & 0.35 & 1.495 & 0 & 0 & 0\\ 0 & 0 & 0 & 0.5919 & 0 & 0\\ 0 & 0 & 0 & 0 & 0.5454 & 0\\ 0 & 0 & 0 & 0 & 0 & 0.4313 \end{array}\right),10^{11}{N/m}^{2}$$

$$e=\left(\begin{array}{cccccc} 0 & 0 & 0 & 0 & 0.332 & 0\\ 0 & 0 & 0 & 0.403 & 0 & 0\\ 0.5428 & 0.707 & 1.636 & 0 & 0 & 0 \end{array}\right),C/m^{2}$$

$$\varepsilon^{S}/\varepsilon_{0}=\left(\begin{array}{ccc} 11.44 & 0 & 0\\ 0 & 11.47 & 0\\ 0 & 0 & 15.47 \end{array}\right),\;\varepsilon_{0}=8.854*10^{-12}F/m$$

**Figure 1:**
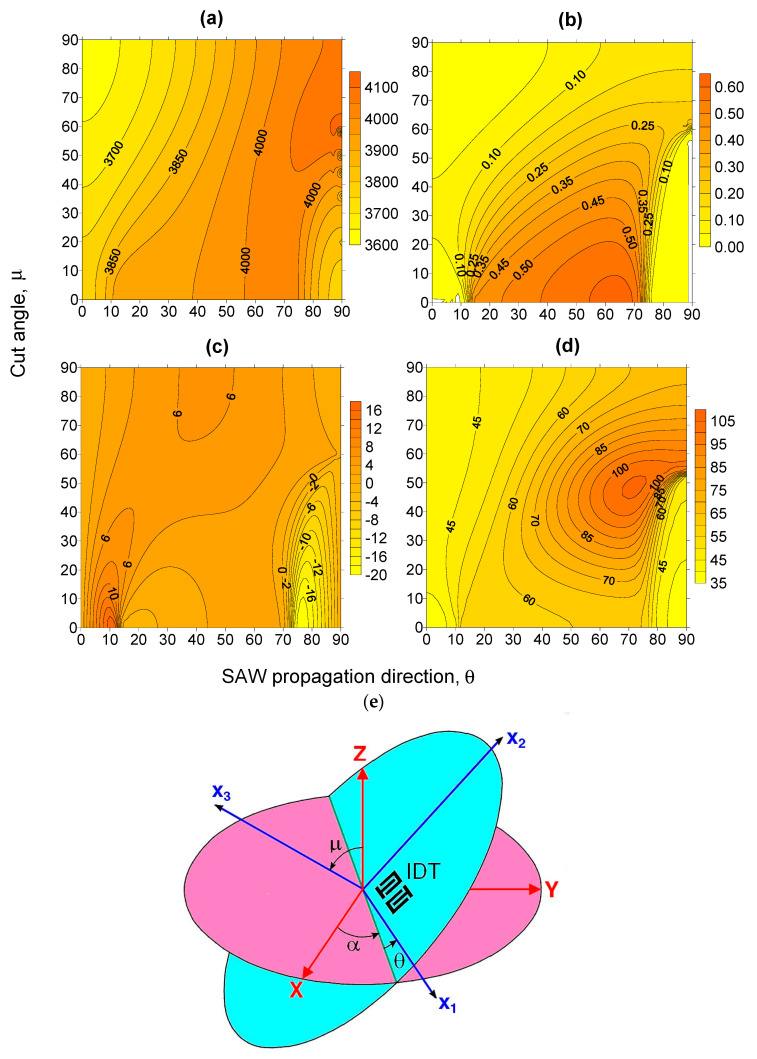
Isolines of SAW parameters as a function of two Euler angles μ and θ in KTP: (**a**) phase velocity (m/s); (**b**) K^2^/2 (%); (**c**) power deflection angle ϕ (degrees); (**d**) TCD (ppm/°C); and (**e**) determining the Euler angle (α, μ, θ) rotation of the Cartesian coordinate system (x_1_,x_2_,x_3_) from the initial crystallophysic orthogonal coordinate axes (X, Y, Z) of the crystal. For Euler angles (0,0,0), the Cartesian coordinate system (x_1_,x_2_,x_3_) coincides with the crystallophysic orthogonal coordinate axes (X, Y, Z) of the crystal.

The KTP crystal mass density was assumed to be 3.024 g/cm^3^ [19]. For aluminium electrodes, the isotropic elastic constants are taken to be as C_11_ = 1.11 × 10^11^ N/m^2^, C_12_ = 0.61 × 10^11^ N/m^2^, and C_66_ = 0.25 × 10^11^ N/m^2^, and the density is ρ = 2.695 g/cm^3^. The SAW parameter computations were performed using our FEM/BEM program (V.1.3, R. Taziev, Novosibirsk, Russia), described in [45]. Our program allows calculating SAW parameters, leaky and bulk wave properties, and simulate effective permittivity, matrix Green function, and the conductance of a finite number IDT electrode structure. In the simulation, the crystal cut is determined by two Euler angles α and μ, and the propagation direction vector is determined by the third Euler angle θ. For angle α = 0°, the crystal cut angle μ and wave propagation direction θ changed in the range from 0 to 90° with the step of 2° (determining Euler angles is provided in Figure 1e).

The SAW properties in piezoelectric materials are described by the system of wave equations [46,47]:

(1) $$\begin{array}{l} \rho \frac{\partial^{2}u_{j}}{\partial t^{2}}-C_{ijkl}\frac{\partial^{2}u_{k}}{\partial x_{i}\partial x_{l}}-e_{kij}\frac{\partial^{2}\varphi }{\partial x_{i}\partial x_{k}}=0,\\ e_{ikl}\frac{\partial^{2}u_{k}}{\partial x_{i}\partial x_{l}}-\varepsilon_{ik}\frac{\partial^{2}\varphi }{\partial x_{i}\partial x_{k}}=0,\;i,j,k,l=1,2,3 \end{array}$$

here φ and u_j_ are the electric potential and mechanical displacement vector, respectively, and ρ is the crystal density. The coordinate system for the SAW problem is taken with the x_3_ outward normal to stress-free substrate surface, and x_1_ is chosen as a wave propagation direction vector. To solve system (1), it is assumed that the solution is a linear combination of partial waves, each of which propagates with the same wave vector k and velocity V along axis x_1_, does not change in direction x_2_ and is attenuated in direction-x_3_ along the substrate depth. The partial waves are taken to be (u_j_, φ)_n_ = (α_j,_ α_4_)_n_exp(ikb_n_x_3_)exp(ik(x_1_ − Vt) with the slowness parameter b_n_ to be chosen so that the partial wave satisfies the wave Equation (1) for a given value of V, where k is the propagation vector. By substituting the solution into Equation (1), eight roots b_n_ for a given velocity V, which are complex-conjugate pairs, are determined. Only four roots b_n_ satisfy the condition of vanishing of each partial wave at large substrate depths. Finally, the unknown velocity V is determined from the boundary conditions at the crystal surface: the vanishing of the normal components of elastic stresses at the free substrate surface and the continuity of the electric displacement or the equality to zero of the electric potential at the crystal surface. A detailed efficient iterative method of searching the velocity is proposed and described in [46,47].

An important SAW parameter is an electromechanical coupling coefficient for piezoelectric materials. It is defined as the percentage difference in the velocity between free surface (V_free_) and stress-free surface coated with an infinitely thin perfect conductor (V_shorted_) [47,48]:

K^2^/2 = (V_free_ − V_shorted_)/V_free_ (2)

This value determines the magnitude of SAW wave generated by the IDT structure.

Another important SAW parameter is the power flow deflection angle (or beam steering angle). The power flow angle is the angle between the power flow vector and the propagation direction vector (phase velocity vector). The power deflection angle ϕ can be found to be as a derivative of phase velocity, with respect to the propagation direction angle θ [47,48]:

(3) $$\tan\phi \;=\;\frac{1}{V}\frac{dV}{d\theta }$$

It is preferable if the angle value ϕ is small or equal to zero for SAW devices [49].

The temperature behavior of some SAW device characteristics is dependent on the temperature change in the delay time between transducers. For SAW resonators and narrow-band SAW filters, it is necessary to have the center device frequency which has low sensitivity to the substrate temperature change [49]. Oppositely, in temperature sensors, it is desirable to have a large device frequency sensitivity to the substrate temperature change with a good linearity [50]. The SAW device parameters sensitivity to the temperature variation are determined by the temperature coefficient of delay (TCD) [48]:

(4) $$TCD\;\;=\;\frac{1}{\tau }\frac{d\tau }{dT}\;=\;\alpha -\frac{1}{V}\frac{dV}{dT}$$

where τ(T) = L(T)/V(T) is the delay time between input and output IDT, L(T) is the distance between two IDT, V(T) is the phase velocity, T is the substrate temperature, and α is the thermal expansion coefficient along the wave propagation direction in the crystal. The delay time τ(T) is calculated using formula τ(T) = L_0_(1 + α(T – 20 °C))/V(T), where L_0_ is the distance between input and output IDT at room temperature 20 °C, α is the thermal expansion coefficient along the wave propagation direction, and V(T) is the phase velocity at temperature T. The phase velocity V(T) is calculated using the material data of elastic, piezoelectric and dielectric constants tabulated in the temperature range from 20 to 140 °C with a step of 20 °C [44]. The temperature dependence of the crystal density is determined by relation ρ(T) = ρ_0_[1 − (α_11_ + α_22_ + α_33_)(T − 20 °C)], where ρ_0_ is the density at room temperature (20 °C). In the simulation (see Figure 1d), the average TCD value of wave was calculated by the formula:

(5) $$TCD\;=\;\frac{1}{\tau }\frac{d\tau }{dT}\;\approx \;\frac{\tau (140\;{}^{\circ}C)-\tau (20\;{}^{\circ}C)}{\tau (20\;{}^{\circ}C)}\frac{1}{120\;{}^{\circ}C}$$

The thermal expansion coefficients of KTP were taken from [19]: α_11_ = 6.8 × 10^−6^/°C, α_22_ = 9.6 × 10^−6^/°C, and α_33_ = −1.3 × 10^−6^/°C.

## 3. Results and Discussion

The SAW-simulated parameters versus two Euler angles μ and θ are presented in Figure 1. It can be seen that the phase velocity changes in the range from 3620 to 4040 m/s. The maximum value of SAW K^2^/2 value is about 0.6%. The SAW piezoelectric coupling coefficients, which are equal to the values between 0.25% and 0.56%, are in the region determined by the Euler angles μ and θ: μ = 0°~60° and θ = 10°~75° (see Figure 1b). The maximum SAW power flow deflection angle value is about 4° in the determined region of K^2^/2 (see Figure 1c). The SAW TCD, which values are in the wide range from 35 to 105 ppm/°C is shown in Figure 1d. It should be noted that the small of SAW TCD (35 ppm/°C) values are in the region of small electromechanical coupling coefficients. Large SAW TCD values in KTP are comparable to the values in the well-known LiNbO_3_ single crystal [48]. As can be seen, KTP does not have orientations with zero SAW TCD values.

**Figure 2:**
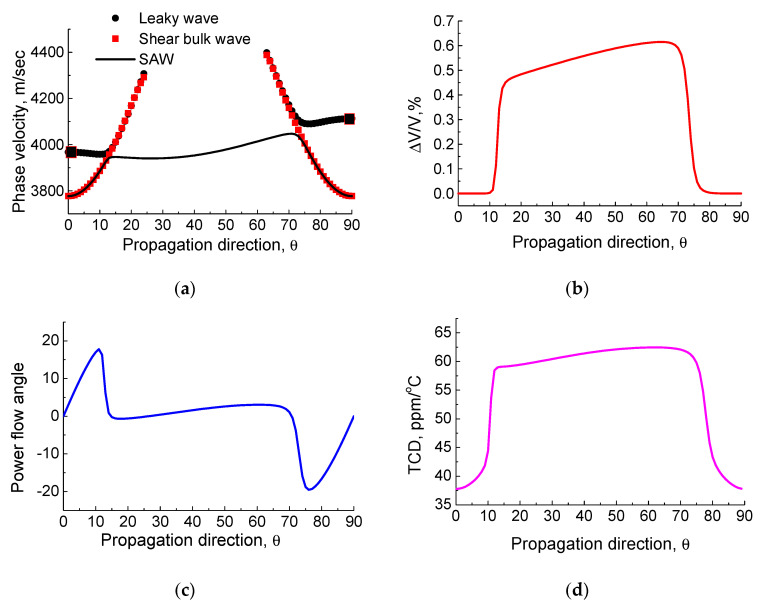
The propagation direction dependence of SAW parameters from the X-axis on the Z-cut of KTP: (**a**) phase velocity; (**b**) ΔV/V; (**c**) power flow deflection angle (degrees); and (**d**) TCD. The dark rectangle denotes the pseudo-surface wave solution.

The SAW velocity, ΔV/V, power flow deflection angle, and TCD versus propagation direction on the Z-cut of KTP are presented in Figure 2. The maximum ΔV/V value is equal to 0.59% along the X + 70°-axis wave propagation direction in the crystal (see Figure 2b). For this propagation direction, the SAW has the phase velocity of 4047 m/s, zero beam steering angle and the TCD of 61 ppm/°C. The dark rectangle in Figure 2a denotes the pseudo-surface wave solution [47]. This wave arises from the leaky wave branch, when the leaky wave propagates along the crystal X-axis. It has an in-plane displacement structure, without the transverse displacement component that causes the leaky wave attenuation. As soon as the wave propagation direction deviates from the crystal X-axis, there is an arising attenuation due to the appearance of the transverse bulk partial component. The pseudo-SAW has the following parameters: V = 3967 m/s, ΔV/V = 0.46% and TCD = 57 ppm/°C. The calculated pseudo-surface wave velocity is in a good agreement with the measured value of ≈3940 m/s [21]. It should be noted that the SAW degenerates into the nonpiezoelectric shear-horizontal (SH) bulk wave along the X-axis propagation direction in the crystal. The calculated SH bulk wave has the velocity value of 3776 m/s.

**Figure 3:**
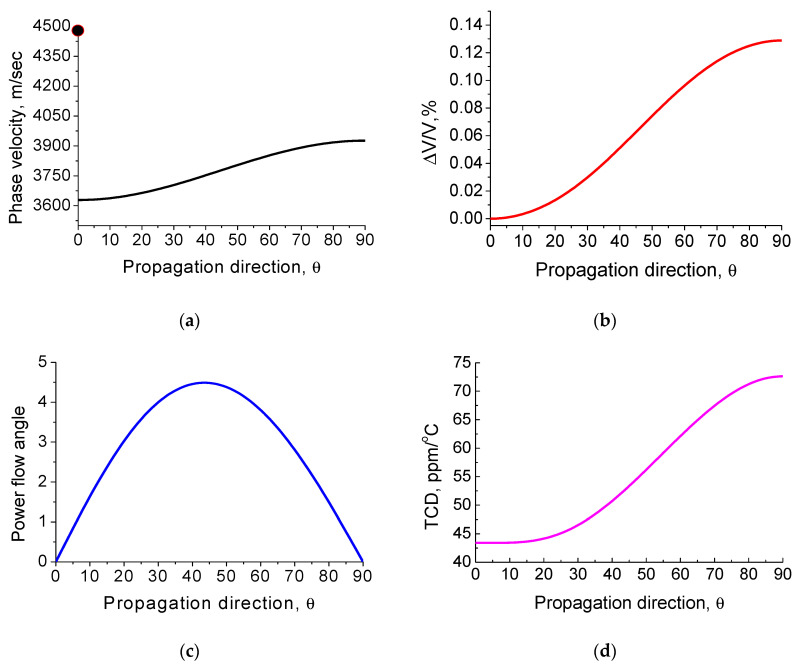
SAW propagation direction dependences from the Y-axis on the X-cut of KTP: (**a**) phase velocity; (**b**) ΔV/V; (**c**) power flow deflection angle (degrees); and (**d**) TCD. The dark circle denotes the BG wave solution.

The SAW velocity ΔV/V, power flow deflection angle and TCD on the X-cut versus wave propagation direction θ on the crystal are displayed in Figure 3. For the X-cut and wave propagation direction along the crystal Z-axis, the SAW has the following parameters: V = 3927 m/s, ΔV/V = 0.13%, and TCD = 72 ppm/°C. The dark circle in Figure 3a denotes the Bleustein–Gulyaev wave solution. The TCD value equals 41 ppm/°C for the BG wave propagation direction along the Y-axis. In the same propagation direction, there exists the nonpiezoactive surface wave, which has a normal u_3_ and longitudinal u_1_ displacement structure similar to those in Rayleigh wave. The main SAW properties for different cuts and wave propagation directions are presented on Table 1.

**Figure 4:**
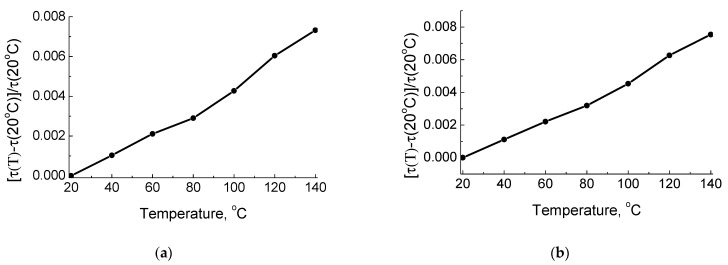
Relative delay time as a function of substrate temperature: (**a**) for the SAW propagation along the X + 70°-axis and (**b**) for the pseudo-surface wave propagation along the X-axis on the Z-cut of KTP.

The relative delay time as a function of substrate temperature with discrete steps of 20 °C for the SAW and pseudo-surface wave on the Z-cut of KTP are shown in Figure 4. As can be seen in Figure 4, the relative delay time slightly deviates from the monotonic linear function of the crystal temperature, thus the approximated Formula (6) can be used for the wave TCD estimation.

**Figure 5:**
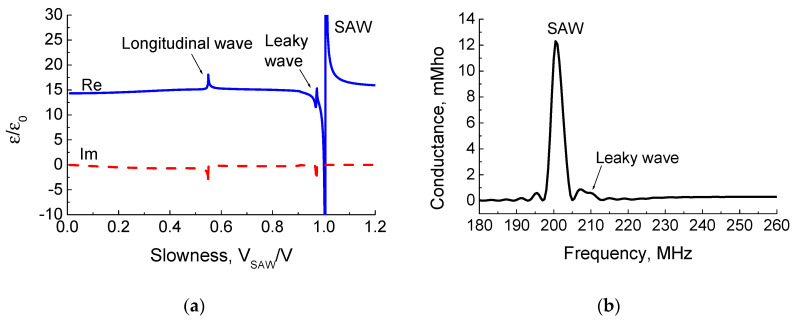
(**a**) Effective permittivity ε_eff_/ε_0_ versus dimensionless slowness V_SAW_/V and (**b**) surface wave IDT conductance response versus frequency for the uniform IDT having 100 Al electrodes with period λ = 20 μm and electrode thickness h/λ = 1% on the Z-cut and the wave propagation direction along the crystal X + 70°-axis.

The effective permittivity versus slowness and the IDT conductance as a function of frequency for the Z-cut and wave propagation direction along the crystal X + 70°-axis are shown in Figure 5. The transducer had 100 Al electrodes with the period of 20 μm and the electrode thickness of 0.2 μm. The transducer aperture was 1600 μm. As can be seen, there is a leaky wave response near the SAW pole. The leaky wave has a phase velocity of about 4173 m/s, an attenuation of 0.14 dB per wavelength, and ΔV/V ≈ 0.072%. The electromechanical coupling coefficient of the leaky wave is eight times smaller than it is for the surface wave. For this reason, its parasitic contribution to the output signal is very small, as can be seen in Figure 5b for the SAW transducer conductance value.

**Figure 6:**
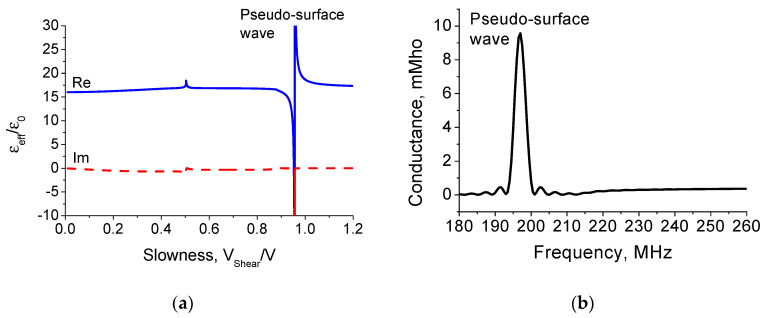
(**a**) Dependence of effective permittivity ε_eff_/ε_0_ versus dimensionless slowness V_Shear_/V and (**b**) IDT conductance as a function of frequency for a homogeneous IDT having 100 Al electrodes with period λ = 20 μm and electrode thickness h/λ = 1% on the Z-cut and the pseudo-surface wave propagation direction along the crystal X-axis.

The effective permittivity versus slowness and the IDT conductance as a function of frequency for Z-cut and the pseudo-surface wave propagation direction along the X-axis on the KTP crystal are illustrated in Figure 6. As can be seen in Figure 6b, there is only the pseudo-SAW excitation on the KTP crystal surface. The IDT had 100 Al electrodes with the period of 20 μm and electrode thickness 0.2 μm. The transducer aperture was 1600 μm.

**Figure 7:**
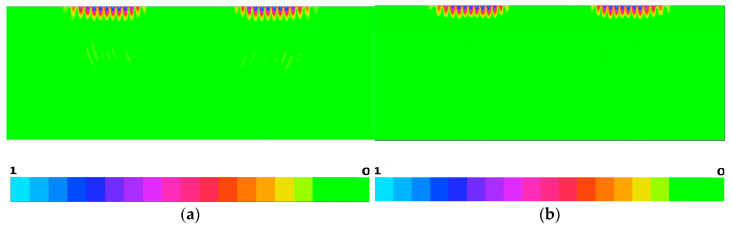
Normalized absolute value of acoustic wave displacement excited by the IDT consisting of five electrode pairs with period λ = 20 μm and thickness h/λ = 1%: (**a**) for the Z-cut and the pseudo-surface wave propagation direction along the X-axis, and (**b**) for the Z-cut and the SAW propagation direction along the X + 70°-axis on KTP.

The normalized absolute values of SAW and pseudo-SAW displacements generated by the IDT consisting of five electrode pairs with periodicity 20 μm in the uniform IDT are illustrated in Figure 7. The transducer is located at the center on the top of Figure 7a,b. The generation source was a harmonic voltage signal with the duration of two periods. For the Z-cut and the wave propagation direction along the crystal X + 70°-axis, the period was taken equal to 1/f_0_ = 1/200 MHz (see Figure 5b). For the pseudo-surface wave propagation direction along the X-axis, it was taken equal to 1/f_0_ = 1/197 MHz (Figure 6b). Here, f_0_ is the frequency of the maximum IDT conductance value in Figure 5 and Figure 6, respectively. It is shown in Figure 6a,b that the excitation of SAW and pseudo-SAW by the IDT flow in a similar way without the large difference in the bulk wave generation on the crystal surface. It can be seen that the IDT spreads a small amount of transducer energy into the bulk acoustic waves, thus propagating into the crystal depths. Both of these propagation orientations are suitable for SAW device applications. The time-domain FEM simulations were performed by the FlexPDE6 software (PDE Solutions Inc., 9408 E. Holman Rd.pokane Valley, WA, USA) [51].

**Figure 8:**
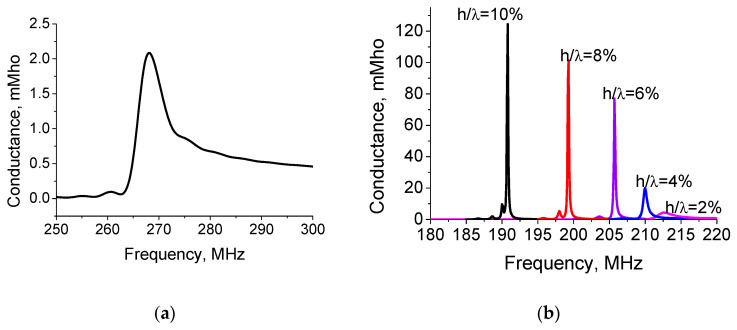
(**a**) Bleustein–Gulyaev wave IDT conductance response versus frequency for uniform IDT with 100 Ti electrodes with period λ = 16 μm and electrode thickness 60 nm; (**b**) for the uniform IDT with 100 Al electrodes with period λ = 20 μm and different thicknesses on the Y-cut of KTP and the wave propagation direction along the crystal X-axis.

The IDT conductance as a function of frequency for the BG wave propagation direction along the X-axis on the crystal Y-cut is shown in Figure 8. The transducer has 100 Ti electrodes with the period of 16 μm and electrode thickness 0.06 μm and acoustic aperture 1250 μm (Figure 8a). In the simulation, the isotropic elastic constants of Ti electrodes are assumed to be C_11_ = 1.57 × 10^11^ N/m^2^, C_12_= 0.75 × 10^11^ N/m^2^, and C_66_ = 0.41 × 10^11^ N/m^2^, and the density is ρ = 4.50 g/cm^3^. The frequency of the maximum of IDT conductance amplitude (≈268 MHz) is in good agreement with the measurements (≈266 MHz) [21]. In Figure 8b, the transducer has 100 Al electrodes with period 20 μm, and the electrode thickness varies from 0.4 to 2 μm. The transducer aperture was 1600 μm. The strong resonance behavior of IDT conductance as a function of electrode thickness was observed. It may be very useful for high-sensitivity liquid viscosity sensor applications. It should be noted that the BG wave has a very small electromechanical coupling coefficient value for infinitely thin electrodes [51]. As the electrode thickness increases, the IDT conductance and electromechanical coupling coefficient of the BG wave also increases [52,53].

**Figure 9:**
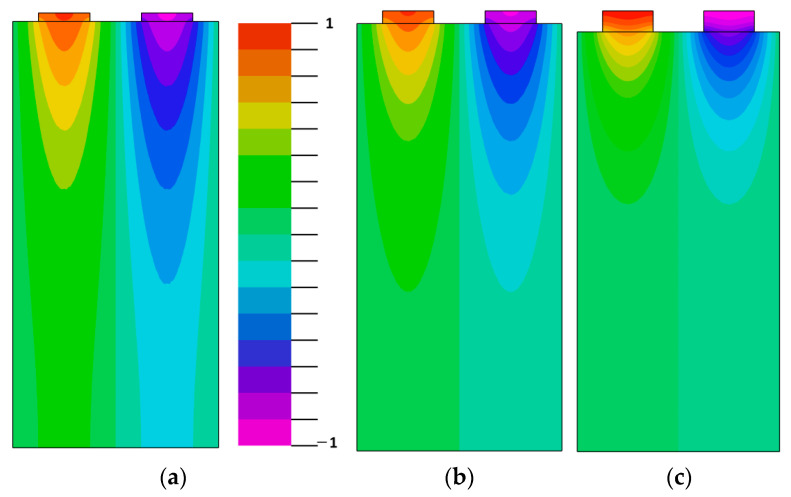
Spatial distribution of normalized amplitude U_2_ into the substrate depth for different IDT electrode thicknesses on the Y-cut and the X-axis wave propagation direction in KTP (calculated by FlexPDE [51]). One period of IDT containing the infinite periodic electrode structure with period λ = 20 μm is shown: (**a**) the electrode thickness h/λ = 4% and resonance frequency f_0_ = 210.6 MHz (resonance frequency f_0_ = 210.0 MHz calculated by our FEM/BEM program [45]); (**b**) the electrode thickness h/λ = 6% and resonance frequency f_0_ = 205.6 MHz (resonance frequency f_0_ = 205.7 MHz calculated by our FEM/BEM program); (**c**) the electrode thickness h/λ = 10% and resonance frequency f_0_ = 190.8 MHz (resonance frequency f_0_ = 190.8 MHz calculated by our FEM/BEM program).

The dependence of shear-wave displacement penetration into the KTP crystal depth, as a function of IDT electrode thickness in infinite periodic structure, is shown in Figure 9. It should be noted that the resonance frequencies obtained by FlexPDE are in the excellent agreement with the results obtained by our FEM/BEM program [45] (Figure 8b). The larger the electrode thickness is, the stronger the Bleustein–Gulayev wave localization to the crystal near-surface region is. It can be seen that most of the BG wave energy is accumulated inside the electrode as the electrode thickness increases. A combined (mixed) wave propagation in the IDT electrode structure can be considered as a superposition of the BG wave and the local shear oscillation mode within the electrode region. This phenomenon is indicated by an increase in the asymmetric conductivity behavior with the increasing electrode thickness (see Figure 8b). As noted above, most of the acoustic energy of this mixed wave is stored on the high-amplitude shear polarization mode confined within the electrode [54,55,56,57,58]. For a large electrode thickness, each IDT electrode oscillates as an elastic resonator coupled to its neighbours by evanescent waves [57,58,59].

**Figure 10:**
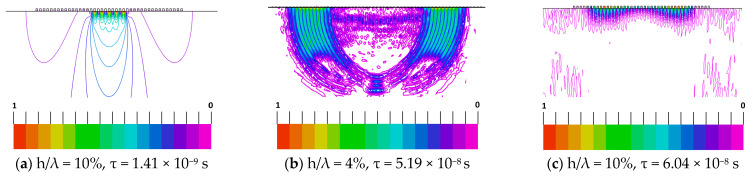
The time dependence of the BG wave excitation by a short harmonic voltage in a homogeneous IDT structure on the Y-cut and the wave propagation direction along the crystal X-axis. The IDT consists of 5 electrode pairs in a periodic structure of 41 electrodes with period λ = 20 μm and various electrode thicknesses h/λ: (**a**) the spatial distribution of electric potential on the electrodes at the excitation time, (**b**,**c**) the amplitude of shear wave displacement distribution for electrode thicknesses, h = 0.08 μm and h = 0.2 μm, respectively. Here, τ is the duration time of wave excitation by the transducer. The FlexPDE software is used for FEM simulations [51].

In Figure 10 are the results of the time-domain FEM modeling of BG wave excitation by five electrode pairs at the center of uniform periodic structure of 41 electrodes with the 20 μm periodicity on the Y-cut and the wave propagation along the X-axis of KTP. The transducer is located at the center on top of Figure 10. The excitation voltage source is the harmonic pulse with a duration of two periods. For the electrode thickness of 0.08 μm, the period was taken equal to 1/f_0_ = 1/198 MHz, and for the electrode thickness of 2 μm, it was taken equal to 1/f_0_ = 1/190.8 MHz, where f_0_ is taken as the frequency of the IDT conductance maximum in Figure 8b. As it can be seen in Figure 10c, the BG wave displacement is highly localized at the crystal surface for the electrode thickness of h= 2 μm (h/λ = 10%). For this electrode thickness, there was a drastic change in the BG wave field structure. The wave is strongly localized in the near-surface region where the electrodes are located. A significant part of the wave energy is in the inner region of the electrodes, and that leads to a strong surface wave slowdown. A short BG wave pulse is strongly slowed down in the transducer structure and the reflection from the electrodes in the transducer induces oscillatory movements inside the uniform periodic structure, radiating its energy by small portions at the ends of the periodic structure. In this case, the BG wave is strongly coupled with the electrode oscillations and becomes as if trapped in the periodic structure of the electrodes and is periodically reflected from them.

It should be noted that such SAW parameters as phase velocity, electromechanical coupling coefficient, power flow deflection angle, and TCD can be measured using standard equipment from the 1970s [48,49,60]. However, a new technique is needed for the investigation of SAW short impulses [61,62,63,64]. A conventional method to study the SAW characteristics is the Laser Doppler Vibrometry (LDV) to measure out-of-plane displacement of wave [65]. However, the BG-type surface acoustic waves have no out-of-plane displacement components within the wave aperture; therefore, new methods should be developed for their detection [66].

In summary, detailed studies of the temperature properties of surface acoustic waves in the KTP crystal showed that there are no cuts and wave propagation directions in which the wave delay time changes weakly with the temperature change, similar to the ST-cut of alpha-quartz (Figure 1d). The SAW TCD values are in the range from 35 to 105 ppm/°C in the KTP crystal, which are of the same order of magnitude as for the well-known LiTaO_3_ and LiNbO_3_ single crystals. KTP possesses sufficiently high SAW coupling coefficients on the Z-cut for the use in SAW devices. For the Z-cut and the wave propagation direction along the X + 70°-axis, it is about 0.59%, and the SAW TCD is equal to 61 ppm/°C. For the Z-cut and the wave propagation direction along the X-axis, the pseudo-SAW has the electromechanical coupling coefficient of 0.46% and the TCD of 57 ppm/°C (Table 1). The pseudo-surface wave and SAW have zero power deflection angle value. The calculated pseudo-surface wave velocity value is in a good agreement with the experimental data [21]. In both KTP crystal cuts, spurious bulk acoustic waves are not generated by the IDT on the crystal surface, and these features make KTP attractive for SAW device applications.

As for the BG waves, their important properties are listed in Table 1. Their electromechanical coupling coefficient is vanishingly small, but it increases rapidly as the transducer electrode thickness increases (Figure 8b). For the Y-cut and the propagation direction along the X-axis, the BG wave, with the increasing electrode thickness in the periodic structure, is strongly localized in the KTP crystal near-surface region. It leads to a combined (mixed) wave propagation is considered as a superposition of BG wave and the local shear oscillation mode within the electrode region. That BG wave behavior can be useful for sensor applications.

## 4. Conclusions

The SAW phase velocity, electromechanical coupling coefficient, power flow deflection angle, and temperature coefficient of delay (TCD) were calculated for different crystal cuts and wave propagation directions in KTP. The isolines of the SAW temperature properties (SAW delay temperature coefficient), which were presented as a function of two Euler angles, show that the SAW has its TCD in the range from 35 to 105 ppm/°C in KTP. These TCD values are comparable to those of well-known LiTaO_3_ and LiNbO_3_ crystals. It was shown that KTP does not have orientations with zero SAW TCD values. Analysis of contour plots of SAW parameters (velocity, electromechanical coupling coefficient, power flow deflection angle, and temperature coefficient of delay (TCD)) showed that SAW has the maximum SAW piezoelectric coupling coefficient value (0.59%) on the Z-cut and the wave propagation direction along the X + 70°-axis. The SAW has the TCD of 61 ppm/°C. For the Z-cut and the wave propagation direction along the X-axis, the pseudo-surface wave has the high coupling coefficient of 0.46%, and its TCD is equal to 57 ppm/°C. The BG wave has TCD values of 35 and 41 ppm/°C on the Y- and X-cuts of KTP, respectively.

The calculated velocities of the pseudo-SAW and BG waves in KTP were in excellent agreement with the experimental data [21]. For the BG wave on the Y-cut and X-axis wave propagation direction, it was shown that, for an electrode thickness ratio to the period of 10%, the BG wave is strongly coupled with electrode oscillations, and it leads to a wave slowdown and strong localization. The short pulse of the BG wave is strongly slowed down inside the transducer structure, and the reflection from the electrodes induces the pulse to make oscillatory movements inside the homogeneous periodic structure, radiating its energy in small portions at the ends of the periodic structure.

## Figures and Tables

**Table 1:** SAW parameters on the different single KTP crystal cuts.

Crystal Cut and Wave Propagation Direction	Wave Type	Velocity, m/s	ΔV/V, %	TCD, ppm/°C
Y,X	BGW	4289	0.018	35
X,Y	BGW	4484	0.037	41
Z,X	PSAW	3967	0.46	57
Z,Y	PSAW	4113	0.66	62
Z,X + 70°	SAW	4047	0.59	61
X,Z	SAW	3927	0.13	72
